# Characterising patients undergoing surgery for lumbar spinal stenosis associated neurogenic claudication in the UK: what does the British Spinal Registry tell us?

**DOI:** 10.1007/s00586-025-09000-x

**Published:** 2025-06-04

**Authors:** Lianne Wood, Rebecca Hunter, Esther Williamson, Khalid M Salem, Opinder Sahota, Bethan E Phillips, Paul Hendrick, Sarah E Lamb

**Affiliations:** 1 University of Exeter, Exeter, UK; 2 Nuffield Department of Orthopaedics, Rheumatology and Musculoskeletal Sciences, University of Oxford, Oxford, UK; 3 Nottingham University Hospitals NHS Trust, Nottingham, UK; 4 University of Nottingham, Nottingham, UK

**Keywords:** Surgery, Lumbar spinal stenosis, Predictors, Registry, Association, Cohort

## Abstract

**Purpose:**

Surgery for lumbar spinal stenosis (LSS) has a variable outcome with many not returning to pre-condition activity levels. We aimed to explore the (1) baseline characteristics of UK patients undergoing surgery, and (2) association of patient characteristics with a clinically important improvement in the 6-month Oswestry Disability Index (ODI) in a population defined by previously developed LSS criteria.

**Methods:**

We used data from the British Spinal Registry (BSR) (2012–2023). Anonymised data included demographics, patient reported outcome measures (PROMs) (ODI; visual analogue scale (VAS) for back and leg pain); quality of life (EQ-5D)) at baseline, and 6-weeks and 6-months post-surgery, surgical procedure, surgery duration and intra-operative blood loss. We used descriptive and multivariate analyses to estimate the association between variables and the minimum clinically important difference (MCID) (30% improvement from baseline) in 6-month disability (ODI). MCIDs define the smallest benefit of value to patients. We explored differences in baseline values between 6-month responders and the total database, and between primary and revision surgery.

**Results:**

In 6801 patients sampled from the BSR the mean age was 70.5 (SD 9.1), 51% (3452/6794) were female, and most were from higher socioeconomic areas (mean Index of Multiple Deprivation rank 84.3, SD 38.8). Preoperatively most had severe disability (ODI 48.7, SD 17.5), moderate leg (VAS 7.0, SD 2.4) and back pain (VAS 6.3, SD 2.5). Proportionally 58% (1160/2008) of the 6-month responders achieved the MCID in ODI. Higher baseline back pain intensity (odds ratio (OR) 0.9, 95%CI 0.9, 0.9), revision surgery (OR 0.5 95%CI 0.3, 0.8), higher 6-week leg pain intensity (OR 0.9, 95%CI 0.8, 1.0) and severe 6-week disability (OR 0.3, 95%CI 0.2, 0.5) reduced the odds of achieving the MCID in 6-month ODI.

**Conclusions:**

Patients undergoing surgery in the UK are severely disabled prior to surgery. The available data suggests that 42% of UK patients do not achieve a clinically important improvement in 6-month disability.

**Supplementary Information:**

The online version contains supplementary material available at 10.1007/s00586-025-09000-x.

## Introduction

Lumbar spinal stenosis (LSS) is the most common reason for people over the age of 65 to undergo surgery [1] and affects ~ 10% of the community-dwelling population [2, 3]. LSS is attributed to degenerative changes causing compression of the spinal nerves within the spinal canal [4]. Most people with LSS experience neurogenic claudication wherein pain, weakness or sensory changes affect the lower limbs, impacting the ability to walk or stand [5].

Globally, there are a total of 9 national spinal registries recording spinal surgical outcomes [6]. These registries, including the British Spinal Registry (BSR), provide an important source of data to understand patient status and surgical outcomes, and can provide valuable information to inform future policy and practice. In the UK, the BSR was set up by the British Association of Spine Surgeons in 2012 to monitor the outcomes of spinal surgery. The BSR collects patient reported outcome measures (PROMs), patient reported experience measures (PREMs), surgical complications, and details of the surgical procedure [7]. The BSR contributes to the National Institute of Clinical Excellence’s clinical guidelines [8]. In addition, registries can provide benefits to patient care by identifying trends and early problems, as well as assessment of specific spinal procedures and related outcomes [6, 9].

PROMs are routinely used to evaluate the effects of treatment, and evaluate outcomes of surgery, usually using self-reported measures to capture domains such as pain intensity and physical function or disability [10–12]. Determining the effect of an intervention such as surgery is often presented as the mean change from pre-intervention to post-intervention. The minimum clinically important difference (MCID) between follow-up and baseline is one method to determine whether the amount of change is clinically useful [13]. The MCID was developed with the intention of incorporating patients’ perceptions of benefit with clinical benefit, to best identify the patients who experience clinically meaningful improvements following surgery. Previous research using spinal registries has defined MCID thresholds for people undergoing surgery for LSS and have found that percentage change or change score were most accurate at reflecting a clinically important change [14].

The aim of this study was to describe the characteristics of BSR patients undergoing surgery for LSS and to estimate the association between patient characteristics and the proportion of patients achieving the MCID in Oswestry Disability Index (ODI) at six months post-surgery as an indicator of early success of surgery.

## Methods

### Study design

This was a retrospective analysis of a prospective cohort of patients registered with the BSR prior to surgery for LSS.

### Patient eligibility

Anonymised patient level data was identified from the BSR for patients over the age of 50 [15, 16] with a diagnosis or code of LSS, as assigned by the surgeon (2/4/2012-28/12/2023). Patients who consented to sharing of anonymised data were eligible, and only patients coded for LSS were included.

### Data collection

Patients are included in the BSR after an invitation from their operating surgeon. Once patients provide pre-operative PROMs data and consent, clinicians input data directly into the BSR (such as patient details, surgical data), following which the BSR contacts the patient for additional PROMs and questionnaires post-operatively. Baseline PROMs are collected in the pre-operative stage from 180 days prior to surgery to 14 days after surgery, 6-week data collection is between 15 and 98 days post-operatively, and 6-month data is collected between 134 and 274 days post-operatively. PREMs collected at 6-weeks after surgery evaluated whether the surgery had achieved their expectations (Likert scale of “much less than expected” to “much more than expected”), helped (Likert scale of “made things worse” to “helped a lot”); and how satisfied they were to spend the rest of their lives with their current symptoms (“very satisfied” to “very dissatisfied”).

### Baseline data

We used PROMs data including ODI scores (scored 0-100), the Visual Analogue Scale (VAS) for back and leg pain (scored 0–10), and the EuroQuol-5Dimension-5 L (EQ-5D) quality of life questionnaire. The EQ-5D included the Health Visual Analogue Score (scored 0-100) and the Index Score for the five domains (scored − 0.59 to 1.00 where 1 is perfect health).

Other baseline data available included year of birth (calculated as age at surgery), sex, presence of reported comorbidities (present/absent, binary value), record of Diabetes Mellitus (yes/no), obesity (BMI ≥ 35) (yes/no), spondylolisthesis (yes/no), smoking (never, stopped or active). To calculate the Index of Multiple Deprivation (IMD) level, BSR registry supplied the outward postcode (i.e., the first part of the postcode) from which we calculated the rank (0-150; a higher score indicates lower socio-economic deprivation) using the English Indices of Deprivation (2019) [17].

### Surgical procedure details

Details on surgical procedures included surgery type (anterior or posterior; revision or primary; addition of fusion or not), duration of surgery (time in minutes), blood loss during surgery (mls) and any complications reported by clinicians post-operatively and/or patients at 6-week follow-up (yes/no).

### Statistical analysis

#### Objective 1 to describe the characteristics of patient undergoing surgery for LSS and outcomes

We described the complete sample of people providing baseline data using descriptive statistics of mean and standard deviation (SD) for continuous variables or counts of frequency and percentage for categorical variables. Pain intensity was interpreted as no pain (0.0 to 0.4), mild (0.5 to 4.4), moderate (4.5 to 7.4) or severe (7.5 to 10) [18]. Severity of ODI scores were interpreted as minimal (0–20), moderate (21–40), severe (41–60), crippled (61–80) and bedbound (81–100) [19]. For each PROM, we calculated the MCID, using recommended calculations and thresholds for LSS [14]. We calculated percentage change scores for VAS and ODI by calculating the absolute change from baseline scores to 6-months, as a percentage of the baseline score. We calculated absolute change scores for the EQ-5D by subtracting baseline values from the follow-up scores [10]. We interpreted these change scores using MCIDs: more than 30% change on the ODI, more than 40% for VAS leg pain, and more than 33% for VAS back pain; absolute change score of more than 0.105 on the EQ-5D index [14] and more than 12 points on the EQ-5D-VAS [20]. We created a binary variable for the ODI baseline and 6-week data (coded as less than 40 (minimal and moderate) or above 41 (severe and worse)) to help with interpretation of the model developed for objective 2.

#### Objective 2 - To evaluate the association between patient characteristics and a clinically important improvement in patient reported physical function at 6-months post-surgery

In a sub-set who provided follow-up data at 6-months post-surgery, we estimated the proportion achieving the MCID in the ODI. We used uni- and multivariate logistic regression, with backward conditional stepwise selection; and an entry and removal threshold of *p* < 0.05 [21]. Patient characteristics included demographics (age, gender, presence of Diabetes and obesity [22]) and pre-operative PROMs (ODI, VAS leg and back pain) for the first, primary model to maximise patient numbers. We then ran a second model including surgical characteristics (primary/ revision surgery, fusion, spondylolisthesis, duration of surgery and blood loss) [15, 23, 24] and a third model including 6-week PROMs data. We did not explore interaction effects between included variables. To meet the assumptions of logistic regression, we assessed the correlation of the independent variables, assessed for multicollinearity (via variance inflation factor using a cutoff of 5 [25, 26]) and a linear relationship between continuous explanatory variables and the logit of the outcome variable [27]. Blood-loss and surgical time both showed non-linear relationships with their logit and were thus transformed and added to the model as the log of the original variable to account for their non-linearity. In addition, we ran a sensitivity model using an ordinal categorisation of the ODI into severity categories [19] Due to the planned multiple testing, we applied a Bonferroni correction and reduced our sensitivity to 0.002 (0.05/33) to reduce a Type 1 error [28]. We used SPSS software (version 28.0.1) for all analyses.

### Missing data

We used complete case analysis. We checked for differences between the baseline values of complete case data and those who provided follow-up data (Tables 1, 2, 3 and 4). We also used a regression analysis on the dependent variable of response at 6 months, using the baseline variables included in our models. In addition, we explored differences between the primary and revision samples (Appendix 3). There were small differences in baseline PROMs (VAS back, leg pain and ODI) between those undergoing primary or revision surgery, but these were not clinically important differences with VAS differences < 1 point on the 10-point scale [29]. ODI differences were 5 points on a 100-point scale, which may indicate a clinical importance [30].

### Ethics approval and consent

Research ethics committee approval was not required for the analysis according to the HRA decision making tool, and patients consented to their data being shared for research purposes on entry into the registry. The data request form was approved by the Steering Committee overseeing BSR management.

## Results

**Fig. 1:**
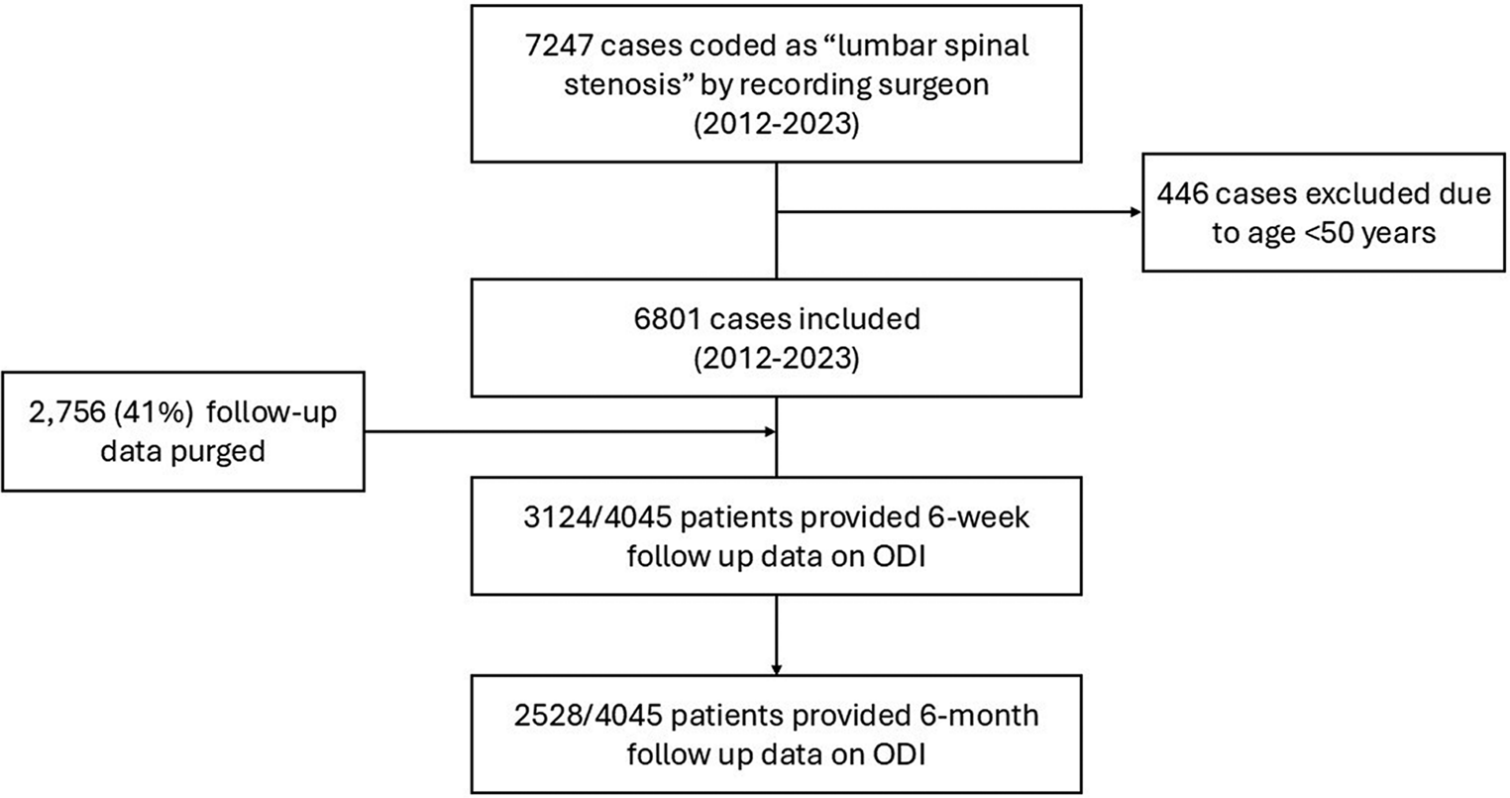
Flow chart to show distribution of patients into study

Of 7,247 BSR registered patients with a diagnosis of LSS between 2012 and 2023, 94% (6,801/7247) were 50 years of age and over and were included in the study (Fig. 1). Follow-up outcomes collection included 59% (4045/6801) and excluded 41% (2,756/6801) due to a lack of consent for follow-up outcomes collection. Follow-up PROM data was provided by 77% (3,124/4,045) at 6-weeks and by 62%(2,528/4,045) at 6-months. Older adults and those with higher baseline disability scores were less likely to respond at 6-months (respectively, OR 0.98; 95% CI 0.96,0.99; *p* < 0.001) and baseline ODI scores (OR 0.99; 95% CI 0.98, 1.00; *p* = 0.02).

### Objective 1 to describe the characteristics of patients undergoing surgery for LSS

**Fig. 2:**
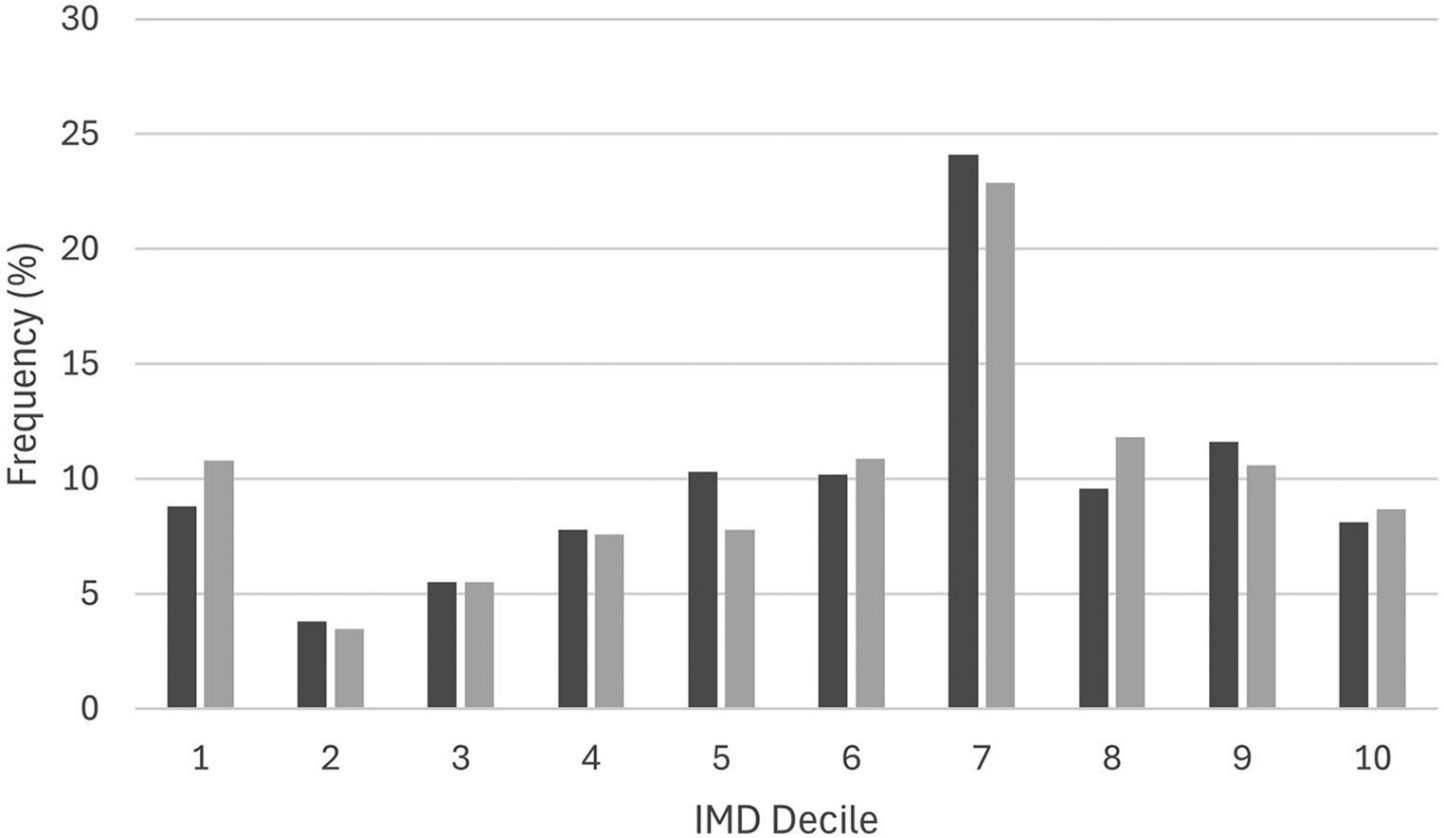
Distribution of IMD deciles by frequency (percentage) for all cases *Where: Dark grey is for all data (n* = *6081) and light grey is for cases providing follow-up data at 6-months post-surgery (n* = *2528) where a higher decile equates to lower socio-economic deprivation. Decile 1: IMD score 0–15*,* decile 2 = IMD score 15–30*,* decile 3 = IMD score 31–45; decile 4 = IMD score 46–60*,* decile 5 = IMD score 61–75*,* decile 6 = IMD score 76–90*,* decile 7 = IMD score 91–105*,* decile 8 = IMD score 106–120*,* decile 9 = IMD score 121–135*,* decile 10 = IMD score 136–150*

Patients were on average 70.5 (SD 9.1) years of age and evenly split across genders (female = 3452/6794, 51%) (Table 1). Patients were disproportionately represented from higher socioeconomic groups with 64% (3439/5403) of patients from IMD scores of 75 and above (Fig. 2). Most surgeries undertaken were primary procedures (5396/6184, 87%), without fusion (4118/5460, 75%), from a posterior approach (5485/5505, 100%), with bilateral midline removed (2970/3702, 80%) (Table 2). Prior to surgery, on average, patients had moderate leg pain (VAS 7.0, SD 2.4) worse than back pain (VAS 6.3, SD 2.5), and severe back pain related disability (ODI 48.7, SD 17.5) (Table 3).

**Table 1:** Baseline patient characteristics of British spinal registry (BSR) patients by follow-up status

	Total BSR Sample (*n* = 6801)		Sub-set providing 6-Month Post-surgery Follow-up (*n* = 2528)	
**Age at surgery** [mean (SD)]	70.5 (9.1)	6801 (100%)	69.4 (8.5)	2510 (100%)
50 years [n (%)]	339 (5%)		119 (5%)	
51-60years [n (%)]	973 (15%)		351 (14%)	
61–70 years [n (%)]	2026 (30%)		757 (31%)	
71–80 years [n (%)]	2429 (36%)		888 (36%)	
81 years + [n (%)]	897 (14%)		352 (14%)	
**Female Gender** [n (%)]	3452 (51%)	6794 (100%)	1294 (51%)	2527 (100%)
**BMI (kg/m**^**2**^**)** [mean (SD)]	28.3 (4.6)	1283 (19%)	28.6 (4.4)	630 (25%)
Underweight (< 18.5) [n (%)]	8 (1%)		0	
Healthy weight (18.5–24.9) [n (%)]	310 (24%)		135 (21%)	
Overweight (25-29.9) [n (%)]	534 (42%)		281 (45%)	
Obese (30+) [n (%)]	431 (34%)		214 (34%)	
**Average Rank IMD** [mean (SD)]	84.4 (38.8)	5403 (79%)	83.7 (40.2)	2052 (82%)
0–30 [n (%)]	684 (13%)		294 (14%)	
31–60 [n (%)]	722 (13%)		268 (13%)	
61–90 [n (%)]	1109 (21%)		380 (19%)	
91–120 [n (%)]	1820 (34%)		715 (35%)	
121–150 [n (%)]	1068 (20%)		395 (19%)	
**Any Comorbidity** [n (%)]	3925 (58%)	6801 (100%)	1485 (59%)	2528 (100%)
Cancer Comorbidity	58 (1%)		20 (1%)	
CVD Comorbidity	674 (10%)		228 (9%)	
Obesity (BMI > 35) comorbidity	968 (14%)		373 (15%)	
Diabetes Comorbidity	778 (12%)		289 (11%)	
Smoker (current or previous)	423 (6%)		180 (7%)	
Other inflammatory comorbidity	188 (3%)		75 (3%)	
Wheelchair dependent	70 (1%)		23 (1%)	
Other medical comorbidity	1949 (29%)		723 (29%)	
**Country of surgery** [n (%)]				
England	5403 (90%)	5999 (88%)	2065 (87%)	2363 (93%)
Wales	461 (7%)		239 (10%)	
Scotland	95 (1%)		52 (2%)	
Ireland	3 (0%)		0 (0)	
Channel Islands	37 (1%)		7 (< 1%)	

**BSR**, British Spinal Registry; **IMD**, Index of Multiple Deprivation; **BMI**, Body Mass Index; **CVD**, cardiovascular disease

**Table 2:** Baseline surgical and diagnostic characteristics of British spinal registry (BSR) patients by follow-up status

	Total BSR Sample (*n* = 6801)		Sub-set providing 6-Month Post-Surgery Follow-up Data (*n* = 2528)	
**Diagnosis** [n (%)]				
Lumbar stenosis	4064 (60%)	6801 (100%)	1507 (60%)	2528 (100%)
Lumbar stenosis and referred pain	117 (2%)		41 (2%)	
Lumbar stenosis and radicular pain	2470 (36%)		928 (37%)	
Lumbar stenosis, referred and radicular pain	150 (2%)		52 (2%)	
**Primary symptom** [n (%)]				
Spinal claudication	1030 (15%)	6801 (100%)	421 (17%)	2528 (100%)
Leg pain	5769 (85%)		2106 (83%)	
Back pain	2 (0%)		1 (0%)	
**Distance walked (m)** [mean (SD)]	159.7 (319.5)	4037 (59%)	161.8 (292.9)	1488 (59%)
**Type of Surgery** [n (%)]				
Primary	5396 (87%)	6184 (91%)	2160 (87%)	2473 (98%)
Revision	788 (13%)		313 (13%)	
**Posterior Surgical approach** [n (%)]	5485 (100%)	5505 (81%)	2078 (100%)	2089 (83%)
**Spinal Fusion** [n (%)]	1,342 (25%)	5,460 (80%)	496 (24%)	2037 (81%)
**Spondylolisthesis present** [n (%)]	2,244 (35%)	6491 (95%)	905 (37%)	2458 (97%)
**Approach** [n (%)]				
Bilateral decompression	2970 (80%)	3,702 (54%)	1117 (80%)	1385 (55%)
Unilateral decompression	732 (20%)		277 (20%)	
**Duration of surgery (mins)** [mean (SD)]	117.7 (117.0)	3802 (56%)	120.2 (174.7)	1382 (55%)
**Blood loss in surgery (mls)** [mean (SD)]	174.7 (273.3)	3701 (54%)	170.7 (206.0)	1343 (54%)
**Clinician reported complication** [n (%)]				
None	530 (74%)	720 (11%)	197 (72%)	273 (11%)
Early	153 (21%)		63 (23%)	
Late	37 (5%)		13 (5%)	

**Table 3:** Patient reported outcome measures at baseline, 6-weeks and 6-months post-surgery: where total BSR sample *n* = 6801 and sub-set providing ODI follow-up at 6-months (complete case) sample *n* = 2528

	Baseline Pre-Surgery		6-weeks Post-Surgery	% Change Score from Baseline to 6-Weeks Post-surgery	6-months Post-Surgery	% Change Score from Baseline to 6-Months Post-surgery	Proportion reaching MCID at 6-Months Post-surgery
	**Total BSR sample**, mean (SD) [n]	**Sub-set providing 6-Month Follow-up**, mean (SD) [n]	**Sub-set providing 6-Month Follow-up**, mean (SD) [n]	**Sub-set providing 6-Month Follow-up**, mean (SD) [n]	**Sub-set providing 6-Month Follow-up**, mean (SD) [n]	**Sub-set providing 6-Month Follow-up**, mean (SD) [n]	**Sub-set providing 6-Month Follow-up**, proportion [n]
**Back Pain (VAS)**	6.3 (2.5) [4448]	6.1 (2.5) [2001]	3.1 (2.6) [1941]	34.1 (131.7) [1561]	3.4 (2.8) [2484]	24.2 (269.8) [1941]	63% [1181/1872]
**Leg Pain (VAS)**	7.0 (2.4) [4464]	6.8 (2.5) [2010]	2.6 (2.8) [1937]	42.1 (296.8) [1569]	3.2 (3.1) [2472]	29.3 (293.5) [1943]	65% [1237/1895]
**Disability (ODI)**	48.7 (17.5) [4491]	46.5 (17.2) [2011]	29.9 (19.8) [1918]	34.3 (51.4) [1573]	29.8 (21.7) [2528]	38.4 (43.9) [2008]	58% [1160/2008]
**EQ-5D VAS**	56.3 (22.6) [4256]	57.1 (22.2) [1973]	70.1 (20.7) [1931]	13.4 (25.1) [1593]	67.5 (22.9) [2453]	11.2 (25.9) [1947]	47% [913/1947]
**EQ-5D Index**	0.4 (0.3) [4212]	0.4 (0.3) [1568]	0.6 (0.2) [1925]	0.3 (0.3) [1190]	0.6 (0.3) [2448]	0.3 (0.4) [1518]	67% [1016/1517]

**VAS-** Visual Analogue Scale; **ODI** – Oswestry Disability Index, **EQ-5D** – EuroQol-5Dimension Questionnaire; **BSR**- British Spinal Registry; **SD**- standard deviation

### Post-operative patient reported outcomes

Post-operative PROMS at 6-months post-surgery were reported in a maximum of 63% (2539/4045)of the sample consented to follow-up. Of the 2528/4045 (62%) providing 6-month ODI follow-up data, 58% (1160/2008) reached the MCID in ODI (Table 3), whilst greater proportion reached the MCID for back pain (63%, 1181/1872), leg pain (65%, 1237/1895), and EQ-5D-index (67%, 1016/1517)] at 6-months. Only health-related quality of life had a smaller proportion of people reaching the MCID [EQ-5D VAS (47%, 913/1947) (Table 3).

Six-week PROMs were for available in 77.2% (3124/4045) of the total baseline sample who consented to collect follow-up PROMs. In the 6-week post-operative PREMs, most reported surgery helped to some degree (1845/2081, 89%) in the 6-week text response questions. However, 41% (844/2101) reported they were dissatisfied or very dissatisfied to spend the rest of their lives with their current symptoms (Table 4).

**Table 4:** Six-week patient reported experience measures by sample follow-up

Patient Reported Values	BSR Sample (*n* = 4045), n(%)		Sub-set providing 6-Month Follow-up (*n* = 2528), n(%)	
**Satisfaction with current symptoms**				
Dissatisfied or very dissatisfied	844 (40%)	2101 (52%)	310 (39%)	789 (31%)
Neither satisfied nor dissatisfied	242 (12%)		87 (11%)	
Satisfied or very satisfied	1015(48%)		392 (50%)	
**Surgery helped or not**				
Made things worse or didn’t help	236 (11%)	2081 (51%)	88 (11%)	781 (31%)
Helped only a little or helped or helped a lot	1845 (89%)		693 (89%)	
**Expectations**				
Much less, less than expected	606 (29%)	2082 (51%)	230 (30%)	781 (31%)
As, more than or much more than expected	1476 (71%)		551 (70%)	
**Complications**,** Yes**	361 (17%)	2073 (51%)	143 (18%)	780 (31%)
**Return to Work**				
Retired/ not working	1228 (67%)	1836 (45%)	466 (69%)	674 (27%)
Returned to work/ Planning to	536 (29%)		188 (28%)	
Not working due to back pain	72 (4%)		26 (4%)	

### Objective 2 - To explore the associations of patient and surgical characteristics with those achieving a minimum clinically important difference on the ODI

In the first, primary analysis (*n* = 1957), we found that more severe baseline back pain reduced the odds of reaching the MCID post-surgery (OR 0.9; 95% CI 0.9, 0.9) (see Table 5).

**Table 5:** Multivariable analysis results for association with reaching MCID in ODI

Achieving the Minimum Clinically Important Change in ODI at 6-months	Odds Ratio (95% CI)
*Patient Characteristics and Baseline PROMs (n* = *1957)*	
Male Gender	1.3 (1.0, 1.4)
Diabetic	1.3 (1.0, 1.7)
Baseline Back Pain Intensity (VAS)	0.9 (0.9, 0.9)**
*Patient Characteristics*,* Baseline PROMs and Surgical Characteristics (n* = *718)*	
Male Gender	1.3 (1.0, 1.8)
Baseline Back Pain Intensity (VAS)	0.9 (0.8, 0.9)**
Revision Surgery	0.5 (0.3, 0.7)**
Fusion performed	1.9 (1.3, 2.7)*
*Patient Characteristics*,* Baseline & 6-Week PROMs and Surgical Characteristics (n = 573)*	
Baseline Severe Disability (ODI > 41/100)	1.9 (1.2, 3.0)**
6-Week Back Pain Intensity (VAS)	0.7 (0.6, 0.8)**
6-Week Severe Disability (ODI > 41/100)	0.3 (0.2, 0.5)**
6-Week Leg Pain Intensity(VAS)	0.9 (0.8, 1.0)**

**ODI**-Oswestry Disability Index; **PROMs**- Patient Reported Outcome Measures; **VAS**- Visual Analogue Scale. *-*p* < 0.05; **-*p* < 0.002

In the second analysis (*n* = 718), we found that undergoing revision surgery (OR 0.5 (95% CI 0.3, 0.7) and more severe baseline back pain (OR 0.9, 95%CI 0.8, 0.9) reduced the odds of achieving the MCID at 6-months post-surgery, whereas undergoing a fusion resulted in a 90% increase in the likelihood of reaching the MCID in ODI (OR 1.9, 95% CI 1.3, 2.7).

Finally, when including 6-week post-surgery outcome measures (*n* = 573), severe disability at baseline (ODI > 41/100) (OR 1.9, 95%CI 1.2, 3.0) increased the odds of achieving the MCID in ODI, whereas worse back pain at 6-weeks (OR 0.7, 95%CI 0.6, 0.8), worse leg pain (OR 0.9, 95% CI 0.8, 1.0) and severe (ODI > 41/100) disability at 6-weeks (OR 0.3, 95% CI 0.2, 0.5) reduced the odds of achieving the MCID in ODI at 6-months post-surgery.

#### Sensitivity analysis

Using the ordinal levels of the ODI baseline and 6-week scores as categories, only the final model demonstrated statistically significant associations across the different levels of ODI. The 6-week ODI scores of moderate [OR 0.4, 95% CI 0.2, 0.6], severe [OR 0.2, 95% CI 0.1, 0.3], and crippling disability [OR 0.1 95% CI 0.0, 0.3] all reduced the likelihood of achieving the MCID. See Appendix 4.

## Discussion

This analysis of BSR data suggests that many patients undergoing surgery for LSS do not achieve clinically important improvement in disability at 6-months post-surgery. Baseline scores demonstrated moderate levels of pain and severe disability, and at 6-weeks, almost half were dissatisfied to live the rest of their lives with their symptoms. The likelihood of achieving a clinically important improvement in disability was influenced by baseline back pain intensity, 6-week post-surgery pain intensity and severe disability scores, revision surgery, and the addition of fusion.

The baseline characteristics of the UK population were different to that reported in Norwegian, Swedish, Danish and Canadian registries. UK registry patients (70.5 years) were older than those in Scandinavian countries (Norway 67.5 years, Sweden 68.9 years, Denmark 68.6 years) [15], and in Canada (64 years) [31]. BSR patients reported higher mean leg pain severity and disability pre-operatively (leg pain 7.0 and ODI 48.6) compared to Norway (leg pain 6.6 and ODI 40), Denmark (leg pain 5.8 and ODI 41) and Sweden (leg pain 6.4 and ODI 44) [15].

In this study, BSR patients had significantly higher rates of comorbidity than Scandinavian countries (54% in UK compared to 25% in Norway, 8% in Sweden and 9.6% in Denmark [15]. Previous reports indicate that comorbidities appear to be underreported [32] and vary in collection form and format across registries [33]. Spine surgeons may have different thresholds of comorbidities deemed relevant for inclusion in spinal registers. In the BSR, the comorbidity field was filled out with substantial variation and included free text and checkbox selection. For example, the “other medical comorbidity” field sometimes referred in the free text to asthma, and in other instances to previous transient ischaemic attacks. In addition, in this dataset, the complications field was poorly reported. Patient self-report at 6-weeks suggested 17% had encountered some form of complication, whereas surgeon-reported complications were far lower at only 3%. Other registry studies have shown a similar pattern with underreporting of complications by surgeons [32, 34]. Without clear quantification and standardisation of patient risk profiles prior to surgery or complications after surgery, it is difficult to assess the impact of these variables on outcomes of interest.

Our study reports slightly smaller proportion of LSS patients achieved the MCID in ODI in comparison to other registers (64% in Norway, 60% in Sweden, 65% in Denmark) [15], with 58% of BSR patients achieving a clinically important improvement. In addition, smaller proportions of those undergoing revision surgery achieved the MCID compared to those undergoing primary surgery in any PROM at 6-months post-surgery despite similar baseline values. This finding is in keeping with other studies, who have suggested a smaller threshold for MCID in those having revision surgery for LSS [23, 35]. This is an important consideration for expectation setting when consenting and planning for revision surgery. MCID thresholds may vary slightly across different populations, such as between those undergoing primary and revision surgery (MCID threshold for the ODI 12.5 for the primary surgery group, 11.8 for the revision surgery group) [23]. In contrast, Glassman et al. [36] reported a change of 18.8 to achieve MCID in ODI scores, for those treated with fusion surgery. However, uncertainty remains about the additional benefits of fusion surgery in this group in comparison to decompression alone [24].

We noticed a mismatch between those achieving a clinically important improvement in pain scores (63% leg pain, 65% back pain) compared to the MCID in disability scores (58%). However, the improvement in pain is in agreement with Scandinavian registers 1-year results (percentage achieving MCID 61–66%) [15]. Longitudinal studies suggest that despite improvements in pain-related disability, patients do not increase their real-life physical activity [37]. This may require further exploration to understand why patients do not increase physical activity despite reduction in pain and improvement in disability.

Severe disability at baseline was associated with increased odds of achieving a clinically important improvement in 6-month disability, whereas 6-week post-surgery pain intensity and disability severity were associated with reduced odds of achieving a clinically important improvement in disability. Other registry studies have shown similar findings with an estimated 11% of patients following an overall poor recovery trajectory at 2-years post-surgery [22, 38]. Other models exploring improvements in pain post-operatively have found that older age, female sex, lower education levels, smoking, quality of life and motor weakness were associated with worse leg pain post-operatively [22]. In addition, obesity and pre-operative pain duration have been shown to be associated with worse short-term improvement in back pain [38].

Surgery is likely to have a greater impact on patients whose baseline symptoms are more severe, allowing greater opportunity for improvement, which has been demonstrated in other studies exploring outcomes for lumbar spinal stenosis patients undergoing fusion and decompressions [39–41]. However, early improvement appears to be related to better overall recovery in the long term, with 6-and 12-week post-operative outcomes associated with long-term prognosis [22, 38]. This suggests that rehabilitation for those with higher pain intensity and disability in the early post-operative phase may be beneficial to support recovery. Long-term pain and disability outcomes appear to be strongly related to early-post-surgery scores, with the greatest improvements post-surgery noted at 3-months post-surgery in a systematic review of 69 studies [42].

### Strengths and weakness

Coverage and completion of LSS surgery cases in the BSR appears to be smaller than expected. This may be due to the selection of eligible cases through a diagnostic code of LSS in contrast to “lumbar decompression surgery”, which sacrificed wider coverage for greater precision in diagnosing LSS. This may have limited the ability of this study to assess baseline predictive features more precisely, or to assess the association with important outcomes such as complication and reoperation rates. However, the lack of standardised diagnostic criteria for the diagnosis of LSS may affect the external validity of this study’s findings. Along with the smaller than expected number of cases, the BSR appears to have a lower response rate to post-operative PROMs than other international registries, which impacted on this analysis plan. However, there were no differences between the sample analysed and those providing baseline data only. The poor response rates may have impacted the findings due to response bias, where those who responded may have performed differently post-operatively than non-responders. A limitation of studies using registry data is that only the variables collected by the registry are available, precluding selection of other, potentially important variables, such as ethnicity or duration of leg and back pain. Accurate diagnostic and procedural coding in surgical and spinal populations is a recognised problem [43], and the small proportion of patients coded as LSS was disappointing.

### Clinical and research implications

We need to find ways to improve patient outcomes from surgery such as improving early post-operative rehabilitation to target early improvement, or prehabilitation to optimise preoperative patient health. Prehabilitation to address cardiovascular optimisation, endocrine diseases and baseline pain levels have shown positive impacts on post-operative outcomes [44–46] and may be a consideration to support this group in waiting well for surgery. Recent evidence suggests a high certainty of prehabilitation programs leading to improvements in pain in patients undergoing lumbar surgery, and moderate-certainty evidence for improvements in health-related quality of life [44]. In addition, we may also wish to consider who has surgery for LSS, as surgery may not provide as much benefit for those with predominant low back pain. Targeted education regarding post-operative expectations such as improvement in leg pain in contrast to back pain may help patients to better prepare for the surgery and recovery period. In addition, educating patients about the importance of national registries and data collection to facilitate better post-operative PROMS completion will improve the quality of BSR data and allow more meaningful interpretation of outcomes.

## Conclusion

Patients undergoing surgery in the UK are severely disabled by symptoms prior to surgery according to our analysis of the BSR data. This study suggests a substantial proportion of patients do not achieve clinically important improvements in disability (as defined by the MCID), despite clinically important improvement in 6-month pain. The primary model suggests that those with higher baseline back pain intensity are less likely to achieve clinically important improvement in disability. In addition, those undergoing revision surgery, with worse baseline back pain intensity, severe 6-week disability, and higher 6-week back pain intensity are less likely to make clinically important improvement at 6-months post-surgery. In contrast, those undergoing fusion, with severe baseline disability have greater odds of achieving clinically important improvement in disability at 6-months. Pre-operative expectation setting may be important to prepare patients for post-operative recovery, as many patients were not satisfied to live with their post-surgery symptoms for the rest of their lives.

## Electronic supplementary material

Below is the link to the electronic supplementary material.

Supplementary Material 1

## Data Availability

Data was provided by the British Spinal Registry under a data sharing agreement. For access to this data, please request access from British Spinal Registry.
