# Supplementary material for: Characterising patients undergoing surgery for lumbar spinal stenosis associated neurogenic claudication in the UK: what does the British Spinal Registry tell us?
Source: Eur Spine J. 2025 Jun 4;34(7):2952–62. doi: 10.1007/s00586-025-09000-x (PMC12367967; doi:10.1007/s00586-025-09000-x)
Supplement: Supplementary file 1 — Supplementary Material 1 [file 586_2025_9000_MOESM1_ESM.docx]

**Appendix 1: Testing the assumptions for logistic regression**

*Table to demonstrate assumption testing for logistic regression: multicollinearity*

| **Variable** | **Tolerance** | **VIF** |
| --- | --- | --- |
| Age | 0.97 | 1.03 |
| Gender | 0.87 | 1.15 |
| Diabetes | 0.97 | 1.03 |
| Obesity | 0.97 | 1.04 |
| Baseline Back Pain Intensity (VAS) | 0.65 | 1.54 |
| Baseline Leg Pain Intensity (VAS) | 0.69 | 1.44 |
| Baseline Disability (ODI) (categories) | 0.56 | 1.78 |
| Surgery Duration | 0.82 | 1.22 |
| Blood Loss | 0.82 | 1.23 |
| Revision Surgery | 0.97 | 1.04 |
| Fusion performed | 0.97 | 1.03 |
| Spondylolisthesis present | 0.90 | 1.11 |
| 6-Week Back Pain Intensity (VAS) | 0.52 | 1.94 |
| 6-Week Leg Pain Intensity(VAS) | 0.58 | 1.73 |
| 6-Week Disability (ODI) (categories) | 0.43 | 2.32 |

Interactions between linearity of continuous predictors and logit

|  | Statistical Significance | OR (95% CI) |
| --- | --- | --- |
| Log Age* Age | 0.71 | 1.08 (0.73, 1.58) |
| *Baseline values* |  |  |
| Log Back Pain *Back Pain | 0.12 | 0.73 (0.49, 1.08) |
| Log Leg Pain * Leg Pain | 0.82 | 1.04 (0.75, 1.44) |
| Log Disability * Disability | 0.71 | 1.01 (0.96, 1.07) |
| *6-week values* |  |  |
| Log Back Pain *Back Pain | 0.05 | 1.39 (1.00, 1.94) |
| Log Leg Pain * Leg Pain | 0.41 | 0.90 (0.69, 1.16) |
| Log Disability * Disability | 0.53 | 0.98 (0.92, 1.05) |
| Log Surgical Time * Surgical time | 0.01 | 1.02 (1.00, 1.05) |
| Log Blood Loss * Blood Loss | 0.02 | 1.00 (1.00, 1.03) |

| **Correlations** | | | | | | | | | | | | | | |
| --- | --- | --- | --- | --- | --- | --- | --- | --- | --- | --- | --- | --- | --- | --- |
|  | | | VAS (Back + Leg) Pain - Baseline Pre-Op | VAS (Back + Leg) - Leg Pain (Worst) - Baseline Pre-Op | ODI Oswestry Disability Index - Score - Baseline Pre-Op | Gender | Age at surgery | Spondylolithesis y/n | Primary/Revision - Code | Surgery_time | Blood-Loss | DM | Obesity | Fusion |
| Spearman's rho | VAS (Back + Leg) Pain - Baseline Pre-Op | Correlation Coefficient | 1.000 | .463^**^ | .461^**^ | -.130^**^ | -.058^**^ | .040^**^ | .045^**^ | .026 | -.006 | -.017 | -.007 | .005 |
|  |  | Sig. (2-tailed) | . | <.001 | <.001 | <.001 | <.001 | .009 | .004 | .207 | .767 | .271 | .623 | .783 |
|  |  | N | 4448 | 4446 | 4328 | 4445 | 4448 | 4210 | 4103 | 2424 | 2367 | 4447 | 4447 | 3565 |
|  | VAS (Back + Leg) - Leg Pain (Worst) - Baseline Pre-Op | Correlation Coefficient | .463^**^ | 1.000 | .494^**^ | -.109^**^ | -.021 | .023 | .047^**^ | .027 | .023 | -.014 | .003 | -.012 |
|  |  | Sig. (2-tailed) | <.001 | . | <.001 | <.001 | .167 | .133 | .003 | .185 | .272 | .360 | .849 | .481 |
|  |  | N | 4446 | 4464 | 4343 | 4461 | 4464 | 4223 | 4118 | 2429 | 2372 | 4463 | 4463 | 3576 |
|  | ODI Oswestry Disability Index - Score - Baseline Pre-Op | Correlation Coefficient | .461^**^ | .494^**^ | 1.000 | -.168^**^ | -.025 | .011 | .078^**^ | .037 | .010 | -.007 | -.014 | .009 |
|  |  | Sig. (2-tailed) | <.001 | <.001 | . | <.001 | .095 | .486 | <.001 | .070 | .635 | .657 | .333 | .587 |
|  |  | N | 4328 | 4343 | 4491 | 4488 | 4491 | 4246 | 4141 | 2457 | 2397 | 4490 | 4490 | 3598 |
|  | Gender | Correlation Coefficient | -.130^**^ | -.109^**^ | -.168^**^ | 1.000 | -.001 | -.248^**^ | .020 | -.019 | .000 | .018 | -.003 | .006 |
|  |  | Sig. (2-tailed) | <.001 | <.001 | <.001 | . | .958 | <.001 | .118 | .235 | .986 | .141 | .797 | .680 |
|  |  | N | 4445 | 4461 | 4488 | 6794 | 6794 | 6485 | 6179 | 3800 | 3699 | 6793 | 6793 | 5454 |
|  | Age at surgery | Correlation Coefficient | -.058^**^ | -.021 | -.025 | -.001 | 1.000 | .023 | -.034^**^ | .008 | -.013 | -.002 | -.013 | -.013 |
|  |  | Sig. (2-tailed) | <.001 | .167 | .095 | .958 | . | .064 | .007 | .610 | .419 | .867 | .279 | .322 |
|  |  | N | 4448 | 4464 | 4491 | 6794 | 6801 | 6491 | 6184 | 3802 | 3701 | 6800 | 6800 | 5460 |
|  | Spondylolithesis y/n | Correlation Coefficient | .040^**^ | .023 | .011 | -.248^**^ | .023 | 1.000 | .000 | .029 | .032 | .006 | .012 | .010 |
|  |  | Sig. (2-tailed) | .009 | .133 | .486 | <.001 | .064 | . | .971 | .083 | .053 | .628 | .352 | .466 |
|  |  | N | 4210 | 4223 | 4246 | 6485 | 6491 | 6491 | 6004 | 3651 | 3549 | 6490 | 6490 | 5219 |
|  | Primary/Revision - Code | Correlation Coefficient | .045^**^ | .047^**^ | .078^**^ | .020 | -.034^**^ | .000 | 1.000 | -.004 | -.017 | -.006 | .010 | .020 |
|  |  | Sig. (2-tailed) | .004 | .003 | <.001 | .118 | .007 | .971 | . | .805 | .318 | .634 | .438 | .159 |
|  |  | N | 4103 | 4118 | 4141 | 6179 | 6184 | 6004 | 6184 | 3491 | 3400 | 6183 | 6183 | 4974 |
|  | Surgery_time | Correlation Coefficient | .026 | .027 | .037 | -.019 | .008 | .029 | -.004 | 1.000 | .475^**^ | -.030 | -.006 | -.012 |
|  |  | Sig. (2-tailed) | .207 | .185 | .070 | .235 | .610 | .083 | .805 | . | <.001 | .063 | .709 | .522 |
|  |  | N | 2424 | 2429 | 2457 | 3800 | 3802 | 3651 | 3491 | 3802 | 3644 | 3802 | 3802 | 3045 |
|  | Blood-Loss | Correlation Coefficient | -.006 | .023 | .010 | .000 | -.013 | .032 | -.017 | .475^**^ | 1.000 | .024 | .007 | .000 |
|  |  | Sig. (2-tailed) | .767 | .272 | .635 | .986 | .419 | .053 | .318 | <.001 | . | .143 | .673 | .999 |
|  |  | N | 2367 | 2372 | 2397 | 3699 | 3701 | 3549 | 3400 | 3644 | 3701 | 3701 | 3701 | 2966 |
|  | DM | Correlation Coefficient | -.017 | -.014 | -.007 | .018 | -.002 | .006 | -.006 | -.030 | .024 | 1.000 | .123^**^ | .011 |
|  |  | Sig. (2-tailed) | .271 | .360 | .657 | .141 | .867 | .628 | .634 | .063 | .143 | . | <.001 | .404 |
|  |  | N | 4447 | 4463 | 4490 | 6793 | 6800 | 6490 | 6183 | 3802 | 3701 | 6800 | 6800 | 5459 |
|  | Obesity | Correlation Coefficient | -.007 | .003 | -.014 | -.003 | -.013 | .012 | .010 | -.006 | .007 | .123^**^ | 1.000 | .015 |
|  |  | Sig. (2-tailed) | .623 | .849 | .333 | .797 | .279 | .352 | .438 | .709 | .673 | <.001 | . | .278 |
|  |  | N | 4447 | 4463 | 4490 | 6793 | 6800 | 6490 | 6183 | 3802 | 3701 | 6800 | 6800 | 5459 |
|  | Fusion | Correlation Coefficient | .005 | -.012 | .009 | .006 | -.013 | .010 | .020 | -.012 | .000 | .011 | .015 | 1.000 |
|  |  | Sig. (2-tailed) | .783 | .481 | .587 | .680 | .322 | .466 | .159 | .522 | .999 | .404 | .278 | . |
|  |  | N | 3565 | 3576 | 3598 | 5454 | 5460 | 5219 | 4974 | 3045 | 2966 | 5459 | 5459 | 5460 |
| **. Correlation is significant at the 0.01 level (2-tailed). | | | | | | | | | | | | | | |

**Appendix 2: Assessment of impact on missingness**

**Regression Analysis:**

Dependent value response at 6-months, Independent variables of baseline values were regressed.

Box Plots to demonstrate baseline values between those responding at 6-months to those not responding at 6-months

1. **Age**


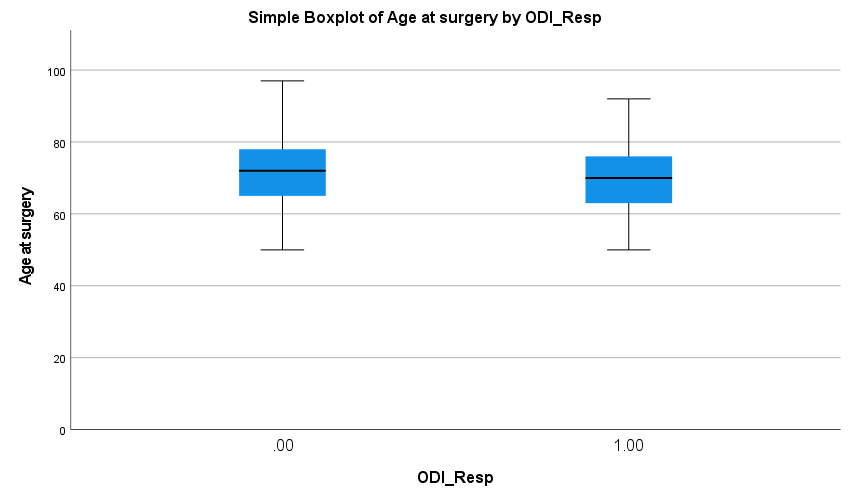


1. **Back Pain Intensity (VAS)**


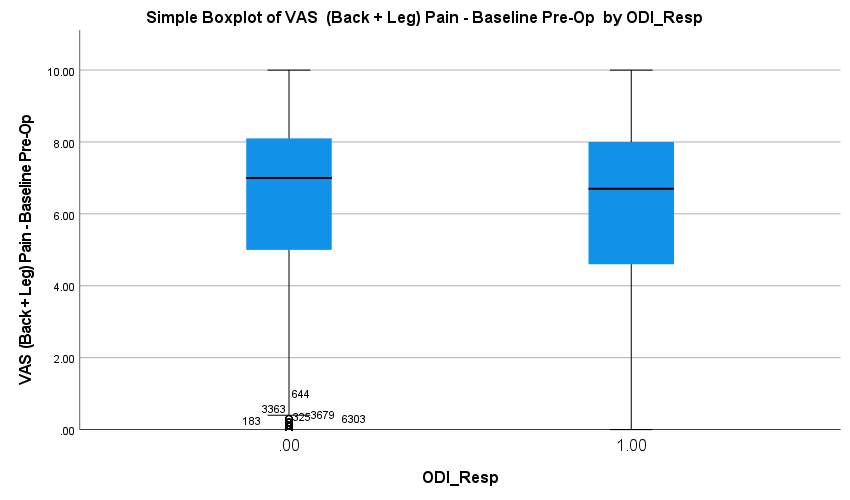


1. **Leg Pain Intensity (VAS)**


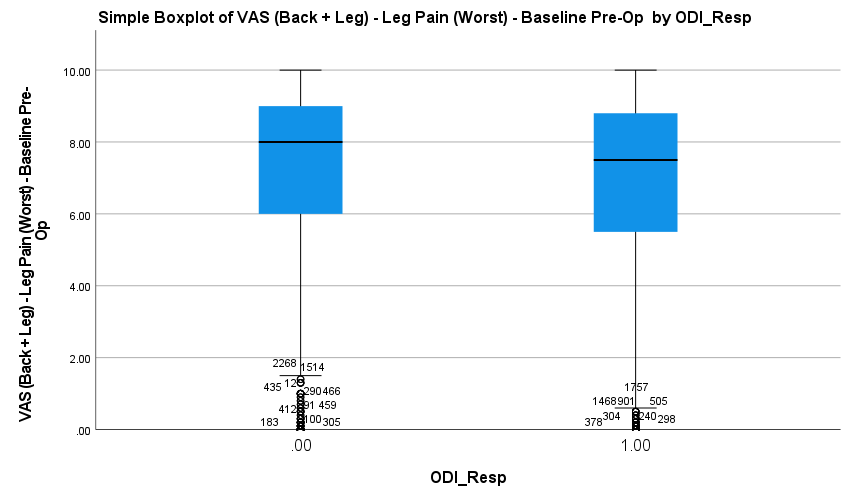


1. **Disability (ODI)**


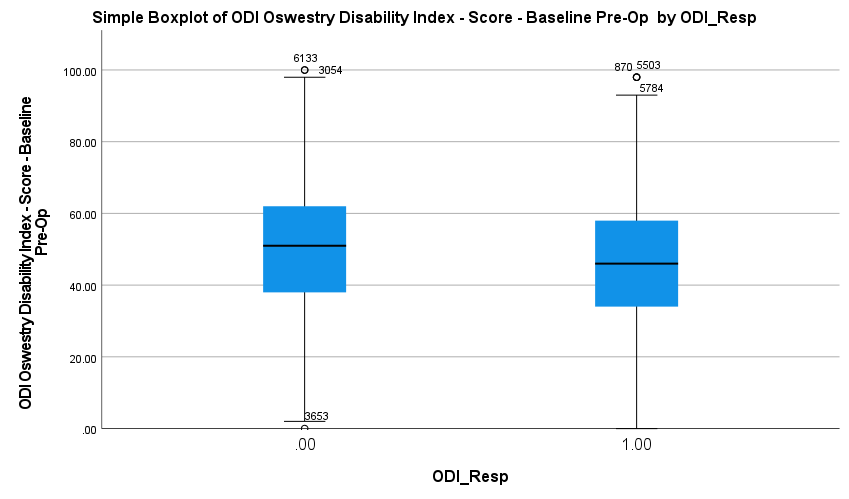


**Appendix 3: Table to show baseline PROMs differences across those undergoing revision or primary surgery procedures in those providing 6-month follow up data**

| Baseline scores | Primary surgery, mean (SD) [n] | Revision surgery, mean (SD) [n] | Mean difference, MD (95% CI) | Proportion reaching MCID at 6mths post-op, % (n) |
| --- | --- | --- | --- | --- |
| Back Pain (VAS) | 6.1 (2.5)  [1728] | 6.2 (2.6)  [236] | -0.2 (-0.5, 0.2) | P: 81% (1348/1671) |
|  |  |  |  | R: 71% (163/230) |
| Leg Pain (VAS) | 6.8 (2.5)  [1737] | 7.2 (2.3)  [236] | -0.4 (-0.7, -0.1) | P: 85% (1418/1665) |
|  |  |  |  | R: 80% (182/229) |
| Disability (ODI) | 45.9 (17.0)  [1737] | 50.9 (17.7)  [236] | -5.0 (-7.3, -2.6) | P: 60% (1031/1734) |
|  |  |  |  | R:44% (104/236) |
| EQ-5D VAS | 57.8 (21.9)  [1707] | 52.4 (23.9)  [240] | 5.4 (2.4, 8.3) | P: 48% (805/1684) |
|  |  |  |  | R: 40% (115/200) |
| EQ-5D Index | 0.4 (0.3)  [1318] | 0.4 (0.3)  [207] | 0.0 (-0.0, 0.0) | P: 68% (878/1287) |
|  |  |  |  | R: 40% (96/238) |

*Boxplot to demonstrate baseline ODI across those undergoing Primary surgical procedures (1) and those undergoing revision surgical procedures (2)*

*
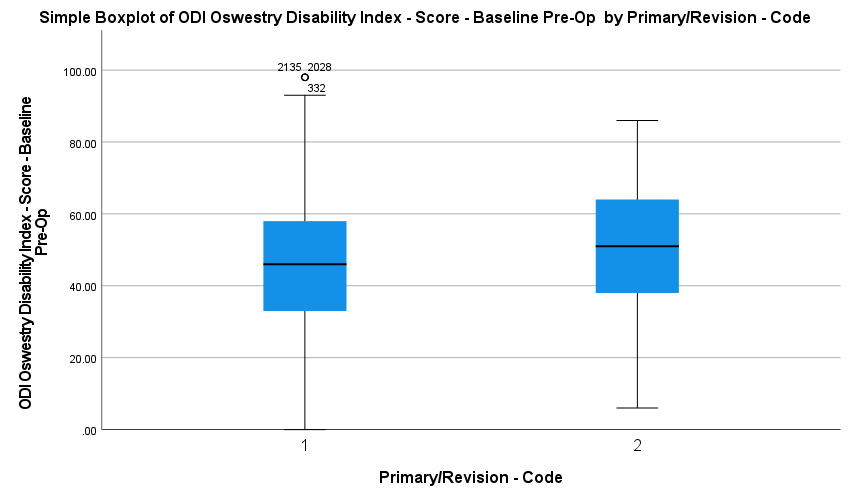
*

*Boxplots to demonstrate the effect of missingness on ODI at baseline in primary surgical procedures (a) and revision procedures (b).*

*a)*

*
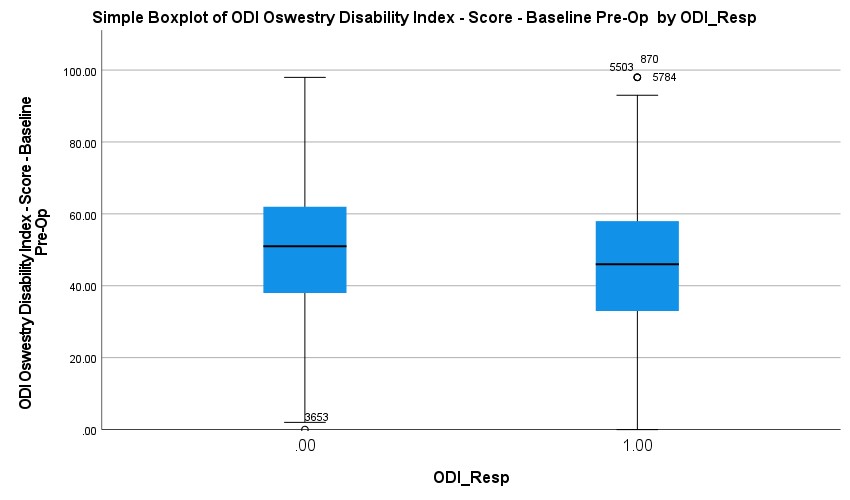
*

*b)*

*
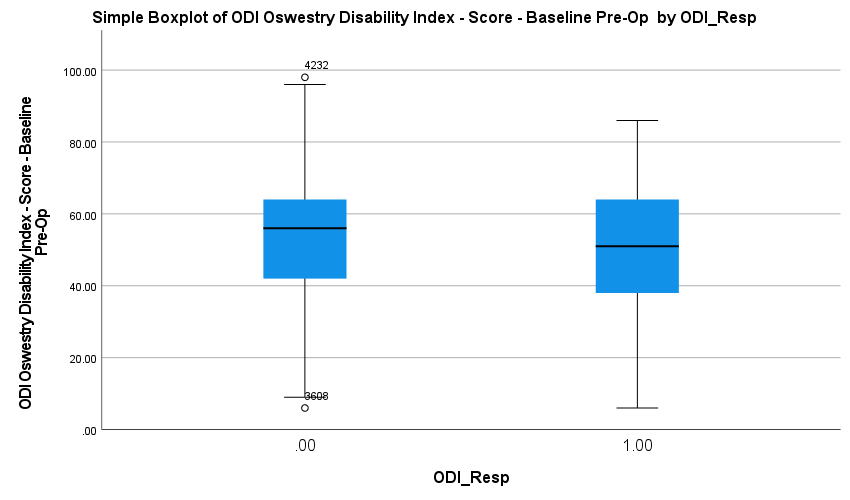
*

**Appendix 4: Table to show Sensitivity Analysis using ODI categorised into ordinal scale of severity (0-20, 21-40, 41-60, 61-80 and 81-100)**

| **Achieving the Minimum Clinically Important Change in ODI at 6-months** | **Odds Ratio (95% CI)** |
| --- | --- |
| *Patient Characteristics and Baseline PROMs (n=1957)* | |
| Male Gender | 1.3 (1.0, 1.4) |
| Diabetic | 1.3 (1.0, 1.7) |
| Baseline Back Pain Intensity (VAS) | 0.9 (0.9, 0.9)** |
| Baseline Crippled Disability (ODI) (61-80) | 0.5 (0.3, 0.8)* |
| *Patient Characteristics, Baseline PROMs and Surgical Characteristics (n=808)* | |
| Male Gender | 1.3 (1.0, 1.7) |
| Baseline Back Pain Intensity (VAS) | 0.9 (0.8, 0.9)** |
| Revision Surgery | 0.5 (0.3, 0.8)** |
| Fusion performed | 1.6 (1.1, 2.2)* |
| *Patient Characteristics, Baseline & 6-Week PROMs and Surgical Characteristics (n=642)* | |
| Baseline Moderate Disability (ODI 41-60) | 2.37 (1.05, 5.35)* |
| 6-Week Back Pain Intensity (VAS) | 0.7 (0.6, 0.8)** |
| 6-Week Moderate Disability (ODI 21-40) | 0.35 (0.21, 0.58)** |
| 6-Week Severe Disability (ODI 41-60) | 0.15 (0.08, 0.30)** |
| 6-Week Crippled Disability (ODI 61-80) | 0.09 (0.03, 0.26)** |
| 6-Week Leg Pain Intensity(VAS) | 0.9 (0.8, 1.0)** |

***ODI****-Oswestry Disability Index;* ***PROMs****- Patient Reported Outcome Measures;* ***VAS****- Visual Analogue Scale. *-p<0.05; **-p<0.002*
